# Characterisation of a Novel Fc Conjugate of Macrophage Colony-stimulating Factor

**DOI:** 10.1038/mt.2014.112

**Published:** 2014-07-29

**Authors:** Deborah J Gow, Kristin A Sauter, Clare Pridans, Lindsey Moffat, Anuj Sehgal, Ben M Stutchfield, Sobia Raza, Philippa M Beard, Yi Ting Tsai, Graeme Bainbridge, Pamela L Boner, Greg Fici, David Garcia-Tapia, Roger A Martin, Theodore Oliphant, John A Shelly, Raksha Tiwari, Thomas L Wilson, Lee B Smith, Neil A Mabbott, David A Hume

**Affiliations:** 1 The Roslin Institute and Royal (Dick) School of Veterinary Studies, The University of Edinburgh, Easter Bush, Midlothian, UK; 2 The University of Edinburgh/MRC centre for Inflammation Research, The Queen's Medical Research Institute, Edinburgh, UK; 3 The University of Edinburgh/MRC Centre for Reproductive Health, The Queen's Medical Research Institute, Edinburgh, UK; 4 Zoetis, Kalamazoo, Michigan, USA

## Abstract

We have produced an Fc conjugate of colony-stimulating factor (CSF) 1 with an improved circulating half-life. CSF1-Fc retained its macrophage growth-promoting activity, and did not induce proinflammatory cytokines *in vitro.* Treatment with CSF1-Fc did not produce adverse effects in mice or pigs. The impact of CSF1-Fc was examined using the *Csf1r*-enhanced green fluorescent protein (EGFP) reporter gene in MacGreen mice. Administration of CSF1-Fc to mice drove extensive infiltration of all tissues by *Csf1r*-EGFP positive macrophages. The main consequence was hepatosplenomegaly, associated with proliferation of hepatocytes. Expression profiles of the liver indicated that infiltrating macrophages produced candidate mediators of hepatocyte proliferation including urokinase, tumor necrosis factor, and interleukin 6. CSF1-Fc also promoted osteoclastogenesis and produced pleiotropic effects on other organ systems, notably the testis, where CSF1-dependent macrophages have been implicated in homeostasis. However, it did not affect other putative CSF1 targets, notably intestine, where Paneth cell numbers and villus architecture were unchanged. CSF1 has therapeutic potential in regenerative medicine in multiple organs. We suggest that the CSF1-Fc conjugate retains this potential, and may permit daily delivery by injection rather than continuous infusion required for the core molecule.

## Introduction

The mononuclear phagocyte system is a family of cells comprising progenitors in the bone marrow (BM), circulating monocytes, and tissue macrophages.^1^ The proliferation, differentiation, and survival of these cells depends upon macrophage colony-stimulating factor (CSF1). Mutations in the *CSF1* locus produce pleiotropic effects on many tissues, reflecting many roles of macrophages in development and homeostasis.^2,3^ A subset of these effects can be mimicked by prolonged treatment with a blocking antibody against the CSF1 receptor (CSF1R).^4^ A *Csf1r*-enhanced green fluorescent protein (EGFP) transgene provides a marker for macrophages in tissues, and enables monitoring of the impacts of treatments that may alter tissue macrophage numbers.^5^ The effects of the blocking antibody supported the concept that macrophage survival/replacement in most tissues, with possible exception of the lung, requires continuous CSF1R signaling.^4,6^

Monocyte and macrophage numbers can be increased above the normal homeostatic levels by CSF1 treatment. Recombinant CSF1 has been tested in clinical trials for several indications,^2^ but has not yet found a clinical application. The original studies when the molecule was cloned focussed on cancer therapy as the indication.^7^ Further studies have been constrained by the cost of the agent. CSF1 has a very short half-life in the circulation of mice (1.6 hours), being cleared by CSF1R-mediated internalisation and degradation by Kupffer cells of the liver.^8^ Renal excretion becomes the major mechanism of clearance when the receptor-mediated clearance is saturated. The 150 amino acid active CSF1 protein produced in bacteria is well below the renal clearance threshold of around 68 kDa (the size of albumin), and consequently the majority of any injected bolus dose is rapidly removed by the kidney. Early studies of human CSF1 actions in mice by Hume et al.^2^ used the large glycoprotein form of the protein produced in mammalian cell culture. It was active with daily injections of 0.5–1 mg/kg. In subsequent studies using the smaller protein expressed in bacteria a 5–10-fold higher dose was required to achieve an increase in circulating monocyte numbers.^2^

When CSF1 was originally identified, it was administered by continuous infusion and was well-tolerated.^7^ The dose-limiting toxicity was thrombocytopenia, which recovered rapidly upon cessation of treatment.^7,9^ Recent studies have reinvigorated interest in CSF1 as a therapeutic agent in tissue repair.^2^ To enable reinvestigation of therapeutic applications of CSF1, especially preclinical evaluations in large animals, we sought to increase the half-life by producing a conjugate with the Fc region of immunoglobulin.^10^ Aside from increasing the molecular size, such conjugates bind the recycling neonatal Fc-receptor, which salvages the protein from endosomal degradation and may allow less frequent dosing of patients. The most studied example is Fc-erythropoietin (EPO), which is in clinical use.^10^ There are several other reports of functional Fc fusion proteins including G-CSF which, like EPO, is structurally related to CSF1. However, the production of active Fc conjugates may be complicated by inappropriate formation of disulphide bonds.^11^ In the case of CSF1, the active protein has three intrachain disulphides and is a disulphide-linked dimer. The additional concern is that a conjugate might potentially link CSF1 to macrophage Fc receptors, which could promote, rather than decrease clearance, or might activate the macrophages in unanticipated ways.^12^

The domestic pig has been used extensively in biomedical research, including preclinical studies of Fc conjugates of EPO.^13^ CSF1 from the domestic pig has the advantage of providing a molecule that is equally active across all mammalian species examined.^14^ We produced a pig CSF1-active fragment conjugated to CH-3 region of pig IgG1a. In this paper we describe the ability of this molecule to drive a massive expansion of tissue macrophage populations in mice. The actions of the molecule *in vivo* led to the surprising conclusion that CSF1 is involved in homeostatic control of the size of the liver and has pleiotropic effects in several other organ systems. These findings expand the potential applications of CSF1 therapy in regenerative medicine.

## Results

### Production and activity of pig CSF1-Fc

**Figure 1:**
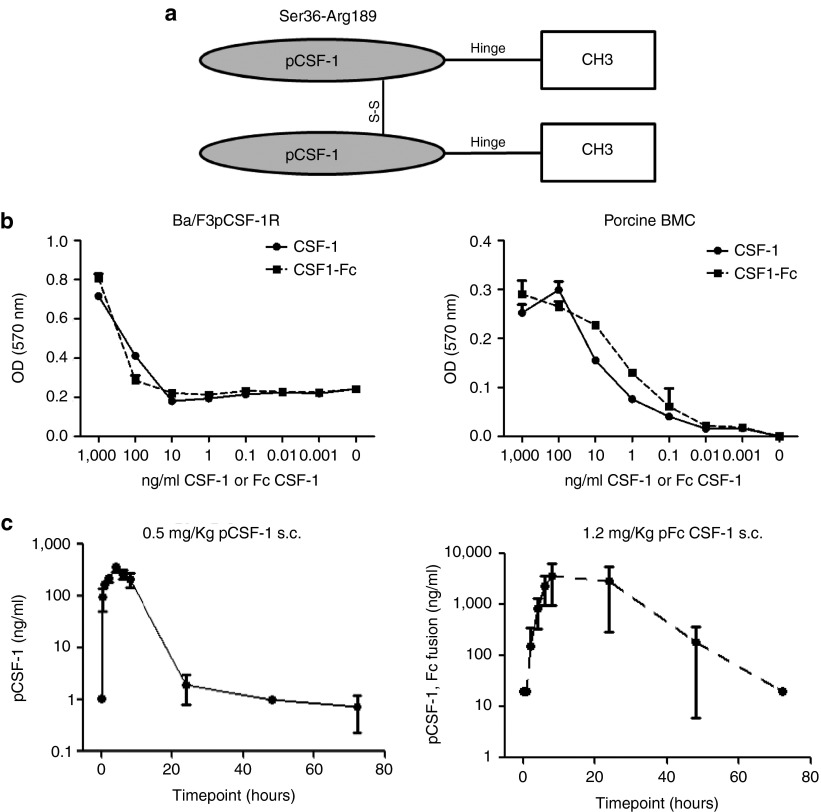
**Pig CSF1-Fc produces viable CSF1-dependent proliferation *in vitro* and has extended plasma half-life *in vivo*.** (**a**) CSF1-Fc molecule was produced by CSF1 joined to the hinge-CH3 region of pig IgG1a. (**b**) CSF1-dependent Ba/F3pCSF1R cells were cultured in rh-CSF1, harvested, washed twice in PBS, and plated for the optimized cell viability assay with either pig CSF1 or pig CSF1-Fc for 48 hours. Pig BM cells were flushed from an adult pig rib and placed in culture with either pig CSF1 or pig CSF1-Fc for 48 hours. Following addition of MTT solution and solubilization, optical density was read at 570nm using a plate reader. Results are the average of triplicate determinations ± SEM from three experiments. (**c**) Three weaner pigs were injected with either 0.5 mg/kg or 1.2 mg/kg pig CSF1 or Fc CSF1-Fc respectively and blood collected at time points above for CSF1 and Fc CSF1-Fc levels to be determined by ELISA. The mean ± SEM is graphed.

A fusion protein comprising pig CSF1 joined to the hinge-CH3 region of pig IgG1a (**Figure 1a**) was expressed in HEK293F cells and purified using Protein A affinity chromatography under contract from Genscript. We have previously demonstrated that pig CSF1 is biologically active on the mouse CSF1R.^14^ The activity of CSF1-Fc was tested in parallel with native recombinant pig CSF1 on the Ba/F3pCSF1R cell assay previously described^14^ and on pig BM cells. The CSF1-Fc protein was equally active on the cell line, and significantly more active on pig BM (**Figure 1b**). Prior to *in vivo* studies, we wished to be certain that the CSF1-Fc did not have any direct macrophage-activating effect, potentially through cross-linking of Fc receptors. Pig BM-derived macrophages (BMDM) were grown in CSF1 as described previously^15^ then treated with pig CSF1-Fc or lipopolysaccharide (LPS). Where LPS produced a massive increase in tumor necrosis factor (TNF) secretion there was no detectable response to CSF1-Fc (data not shown). To test the effect of the Fc conjugate on clearance, pig serum samples were collected at various time points following subcutaneous injection of either CSF1-Fc or CSF1 and assayed using an anti-CSF1 antibody ELISA developed in-house (**Figure 1c**). As anticipated, the administration of CSF1-Fc achieved a 10-100-fold higher peak concentration than unconjugated CSF1 alone, and an elevated concentration was maintained for up to 72 hours.

Previous studies in humans and primates have indicated that CSF1 is relatively well-tolerated.^7,16^ However, the Fc conjugate could produce a secondary stimulus. An initial study indicated that a daily dose of 0.5 mg/kg was sufficient to induce a twofold to threefold increase in total leukocytes after 3 days, and also produced substantial increases in tissue histiocytes in the liver (not shown). We subsequently treated a cohort of 13 newborn piglets with 0.5 mg/kg every second day for 2 weeks, and sacrificed them 2 weeks later. Animals were weighed and monitored continuously, and blood taken at days 1, 7, 13, and 24. As observed in patients treated with recombinant CSF1^7,9^, there was an increase in total white blood cells (WBC), which was not restricted to monocytes. Total WBC and total lymphocytes were increased transiently even in the control piglets; this was extended by the CSF1-Fc treatment. All of the WBC populations declined following the cessation of treatment. There was no evidence of increased temperature or behavioral changes during the treatment period, and all animals gained weight rapidly (**Supplementary Figure S1**). In summary, the CSF1-Fc conjugate appears active, safe, and well-tolerated in a large animal.

### CSF1-Fc expands macrophage populations in blood and organs

**Figure 2:**
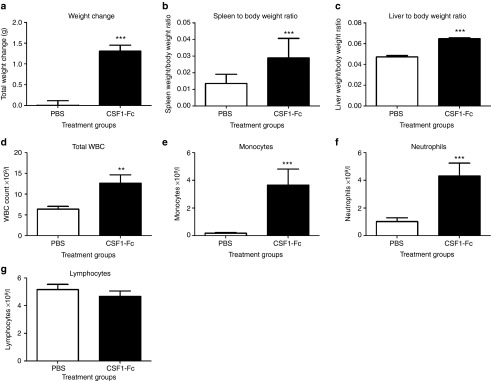
**Effect of CSF1-Fc on body weight, organ weights, and white blood cell counts.** Mice were injected with PBS or 1 µg/g pig CSF1-Fc for four days prior to sacrifice on day 5. Blood was collected into EDTA tubes post-mortem and complete blood count assessment performed. Graphs show the mean ± SEM. Significance is indicated by *P < 0.05, **P < 0.01, ***P < 0.001 using a Mann–Whitney test. *n* = 20 mice per group for weights and *n* = 12 mice per group for blood cell counts (**a**) Body weight was recorded before each injection. Total body weight change over the duration of the experiment was graphed (**b**) Spleen/body weight ratio (**c**) Liver/body weight ratio (**d**) Total WBC count (**e**) Monocyte number (**f**) Neutrophil number (**g**) Lymphocyte number. PBS, phosphate-buffered saline.

As noted in the introduction, the effects of CSF1 mutation in mice suggest that CSF1-dependent macrophages have many roles in homeostasis. Notwithstanding the apparent safety, side effects that have not been considered could constrain therapeutic applications of a much more active form of CSF1. To test the effect of CSF1-Fc in more detail in mice, we first performed a dose-response study. We treated daily to maintain continuous elevation of CSF1, since there is some evidence of decline in levels after 24 hours. It may be that less frequent treatment would produce the same outcome, but this has not been evaluated systematically. A series of 4 daily treatments with CSF1-Fc produced a maximal increase in blood leukocytes at 0.5 and 1 mg/kg. Administration of 1 mg/kg of recombinant pig CSF1 produced no detectable increase in circulating leukocytes or tissue macrophages, despite the equivalent activity in the *in vitro* assays (data not shown). We therefore used the dose of 1 mg/kg for subsequent studies. By contrast to many previous studies, and in the light of the known roles of CSF1 in both male and female fertility,^3^ we examined equal numbers of male and female mice. There was a significant increase in total body weight in the CSF1-Fc treated group (**Figure 2a**). The most obvious effect of the CSF1-Fc was hepatosplenomegaly, which was visibly evident upon necropsy, and which accounted for almost all of the body weight gain. Administration of CSF1-Fc doubled the spleen/body weight ratio (**Figure 2b**) and increased the liver/body weight ratio by 50% (**Figure 2c**). There was no difference in gross kidney or lung weight or organ/body weight ratios. The total WBC count was significantly increased in mice treated with CSF1-Fc, mainly due to monocytosis and neutrophilia (**Figure 2d**–**g**).

**Figure 3:**
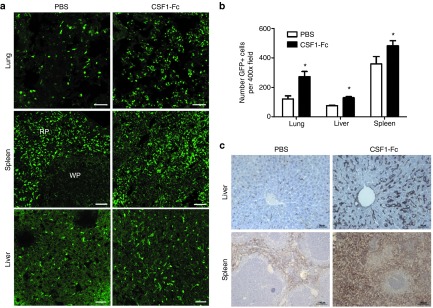
**Effect of CSF1-Fc on tissue macrophage populations.** Mice were injected with PBS or 1 µg/g pig CSF1-Fc for four days prior to sacrifice on day 5. (**a**) Organs from *Csf1r*-EGFP^**+**^ mice were removed post-mortem and frozen in OCT prior to cutting and examination of EGFP^**+**^ cells. Tiled images of 3 × 3 400x field of view were generated. Bar = 50µm RP, red pulp; WP, white pulp. (**b**) The total number of EGFP^**+**^ cells were calculated along with mean total fluorescence ± SEM from five representative images/mouse/organ. A Kruskal–Wallis test with Dunn's multiple comparison test was performed with significance set as *P < 0.05, **P < 0.01, ***P < 0.001. (**c**) Formalin fixed liver and spleen tissue was prepared and stained for F4/80. *n* = 12 mice per group. PBS, phosphate-buffered saline.

The *Csf1r*-EGFP^+^ MacGreen reporter mice^5^ provides a unique tool to monitor the response to CSF1-Fc. The increased numbers of EGFP^+^ cells in the lung, spleen, and liver (**Figure 3a**) after CSF1-Fc treatment was so great that it could be detected as a global increase in total fluorescence (**Figure 3b**). Increased macrophage numbers were confirmed by F4/80 immunostaining for both liver and spleen (**Figure 3c**). The effect of CSF1-Fc in the lung was unexpected. Prolonged treatment of mice with anti-CSF1R antibody was shown to deplete alveolar macrophages, but not interstitial macrophages.^6^ In the lung of CSF1-Fc treated mice, there was a twofold to threefold increase in EGFP^+^ cells that appeared to be confined to the interstitium. The increased numbers and diffuse infiltration of EGFP^+^ cells within the spleen of CSF1-Fc treated mice was so extensive it was impossible to identify the boundaries of the red and white pulp, implying that there was extensive infiltration of the lymphoid follicles by EGFP^+^ cells (**Figure 3a**,**b**). In the liver, the EGFP reporter gene is expressed solely in Kupffer cells which constitute about 8% of the total liver cell population.^5^ The relative proportion of EGFP^+^ cells was increased around twofold in the CSF1-Fc-treated mice (**Figure 3a**,**b**). The location of the positive cells was unchanged, and was consistent with the sinusoidal location of Kupffer cells (**Figure 3c**).

### Pleiotropic effects of CSF1-Fc treatment

**Figure 4:**
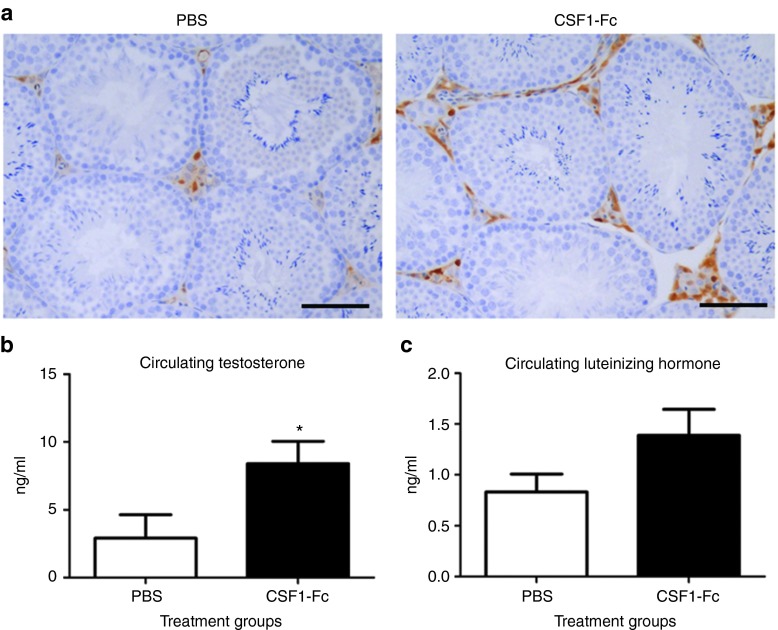
**Effect of pig CSF1-Fc on male reproductive tract.** Mice were injected with PBS or 1 µg/g pig CSF1-Fc for four days prior to sacrifice on day 5. Serum was collected as well as tissue, which was fixed and processed as described in materials and methods. Graphs show the mean ± SEM. Significance is indicated by *P < 0.05 using unpaired *t*-tests. (**a**) Representative sections show macrophages within the testicular interstitium, stained with a macrophage marker, Mac2. Bar = 50µM. (**b**) Serum levels of testosterone and (**c**) Luteinizing hormone.

**Figure 5:**
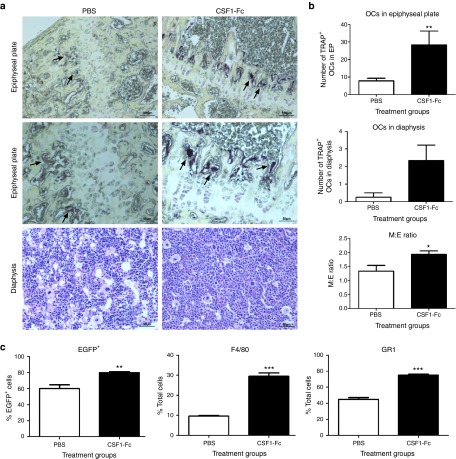
**Effect of pig CSF1-Fc on BM cells and bone.** Mice were injected with PBS or 1 µg/g pig CSF1-Fc for four days prior to sacrifice on day 5. The right femur from each mouse was harvested and prepared for histological examination. All graphs show the mean ± SEM. Significance is indicated by *P < 0.05, **P < 0.01, ***P < 0.001 using a Mann–Whitney test. (**a**) TRAP IHC was performed. Black arrows represent OCL in epiphyseal plate. The number of positive cells in each section was counted. *n* = 4 mice/group. (**b**) The myeloid:erythroid ratio was determined and graphed. *n* = 4 mice/group. (**c**) BM cells were flushed from the femurs post-mortem and prepared for FACS as described in materials and methods. The percentage of F4/80 and Gr1 cell populations were determined by exclusion of dead cells using Sytox blue. *n* = 12 mice/group.

The close physical and functional interaction between testicular interstitial macrophages (TIMs) and Leydig Cells is essential for normal testis function. TIMs have been demonstrated to be associated with development and function of LC and CSF1 has also been implicated in the control of male fertility and testosterone production.^17^ To preserve the histology of the testis for this application, the mice were perfusion fixed which decreases EGFP detection. Macrophages were localized in the testis of MacGreen mice injected with CSF1-Fc using anti-GFP antibody and confirmed using the Mac2 antibody, which detects galectin-3, and the anti-macrophage ER-HR3 antibody (data not shown). There was a clear increase in interstitial macrophage numbers in CSF1-Fc-treated animals compared to controls (**Figure 4a**). To assess the consequence of these changes, we examined the circulating levels of testosterone and luteinizing hormone (LH), which participates in a physiological feedback loop with testosterone, in the blood. The CSF-1-deficient *op/op* mouse was reported to have depleted levels of both testosterone and LH, indicating a disruption of the hypothalamic feedback loop.^17^ We demonstrated a significant increase in circulating testosterone, with no difference in circulating LH, in CSF1-Fc-treated animals compared to phosphate-buffered saline (PBS) controls (**Figure 4b**). CSF1 has been inferred to have direct effects on epithelial cell proliferation and differentiation in the intestine. The Paneth cells of the crypt appear to be depend upon CSF1 signaling, and are absent from the CSF1-deficient *op/op* mouse.^18^ The MacGreen reporter gene is not detected on any cells within the epithelium in the intestine, including the crypts and Paneth cells, but there are large numbers of EGFP^+^ cells in intimate contact with the underlying basement membrane (**Supplementary Figure S2a**). The lamina propria of MacGreen mice contains a very dense network of EGFP^+^ cells.^5^ Against this high background, we did neither detect any increase in EGFP^+^ cells following CSF1-Fc treatment, nor any overt change in villus thickness or architecture. Cryosections were immunostained to detect the Paneth cell marker, lysozyme in intestinal crypts. There was no difference in apparent numbers, location, or staining intensity between the control and CSF1-Fc treated samples, nor any effect on villus architecture or morphology to suggest impacts on epithelial proliferation (**Supplementary Figure S2b**). Together with RANK ligand, CSF1 can activate multiple intracellular signaling pathways in osteoclasts.^2,19^ The administration of CSF1-Fc caused a clear increase in the number of TRAP^+^ osteoclasts within the epiphyseal plate compared to PBS control mice (**Figure 5a**,**b**). Within the BM, there was a significant increase in the myeloid: erythroid ratio from the normal range of 1.3–1.5 to a ratio of 1.8–2.0 (**Figure 5b**). CSF1 treatment of mice was reported to increase the numbers of CSF1 responsive cells within the BM^20^, consistent with more recent evidence that it has a direct instructive role on progenitors.^21^ Marrow cells were also stained with the macrophage-specific antibody F4/80 and anti-Ly6C/G (Gr1). CSF1-Fc caused a large increase in the proportions of marrow cells that were EGFP^+^, F4/80^+^, and Gr1^+^ (**Figure 5c**).

### The origin of the increase in liver and spleen weight in CSF1-Fc treated mice

**Figure 6:**
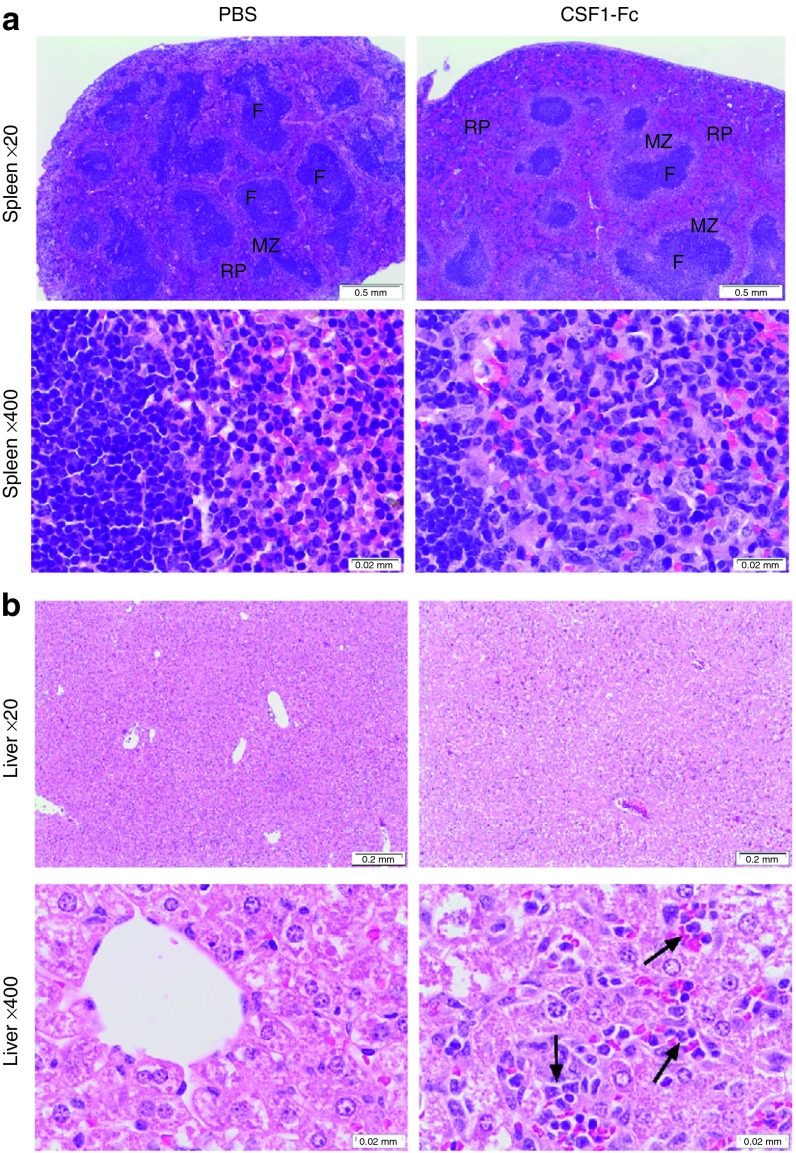
**Effect of pig CSF1-Fc on liver and spleen pathology.** Mice were injected with PBS or 1 µg/g pig CSF1-Fc for four days prior to sacrifice on day 5. The spleen (**a**) and liver (**b**) were removed post-mortem and placed in 10% formal saline prior to sections being cut and stained with H&E for blind histological examination. Representative images are shown. F, follicle; MZ, marginal zone; RP,red pulp. Arrows represent sinusoidal infiltrate.

**Figure 7:**
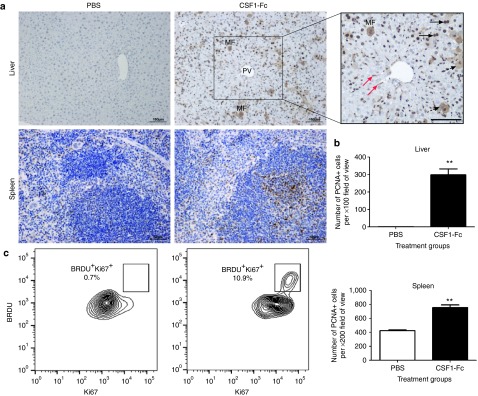
**Effect of pig CSF1-Fc on liver PCNA and Ki67 as markers of proliferation.** Mice were injected with PBS or 1 µg/g pig Fc CSF1-Fc for four days prior to sacrifice on day 5. (**a**) Following harvest of organs and preservation in 10% formal saline, 8 µm sections were cut and PCNA immunohistochemistry performed. Slides from each mouse were examined for the presence of PCNA^+^ cells (brown color) and a representative image is shown. Black arrow, bi-nucleate hepatocyte; dashed arrow, cytoplasmic PCNA staining; red arrow, Kupffer cells; MF, mitotic figure; PV, portal vein; Bar = 100µm. (**b**) The mean number of PCNA^+^ cells ± SEM is graphed. Significance is indicated by *P < 0.05, **P < 0.01, ***P < 0.001 using a Mann–Whitney test. (**c**) Following PBS perfusion, the liver was removed, digested and non-parenchymal cells were purified. Live CD45^+^F4/80^+^ cells were stained for BRDU and Ki67.

In the spleen, the majority of the increase in size was attributable to increased red pulp, and also to expansion of the marginal zones (**Figure 6a**). In the liver, the sinusoidal macrophage numbers were substantially increased. There was no evidence of hemostasis, no infiltration by other leukocytes such as neutrophils that would indicate tissue damage, nor of apoptosis of hepatocytes (**Figure 6b**). Histological examination revealed numerous mitotic figures in hepatocytes in the treated mice, where they were absent from controls. Accordingly, sections of liver, spleen, lung, and kidney were stained for proliferating cell nuclear antigen (PCNA). There was a significant increase in the number of PCNA^+^ cells in the liver and spleen of CSF1-Fc treated mice (**Figure 7a**,**b**). In the liver, the majority of PCNA^+^ cells were hepatocytes (**Figure 7a, black arrow**), but the PCNA^+^ cells within the sinusoids resembled Kupffer cells (**Figure 7a, red arrow**). Both nuclear and cytoplasmic PCNA staining was identified in the treated mice livers (**Figure 7a, dashed arrow**). A transition from cytoplasmic to nuclear PCNA staining in hepatocytes is also observed in regenerating liver.^22^ PCNA^+^ cells were distributed throughout the parenchyma. The same pattern of widespread hepatocyte proliferation is seen after partial hepatectomy, where new hepatocytes derive from preexisting hepatocytes rather than stem cells.^23^

CSF1-Fc can drive recruitment of cells to the peritoneal cavity, including both Ly6C^+^ monocytes and granulocytes, and many of the infiltrating cells are actively proliferative.^24^ To confirm the apparent proliferative capacity of the macrophages in the treated liver, we disaggregated the livers of control and CSF1-Fc treated mice following labeling with bromodeoxyuridine (BrdU), and examined the phenotype of the macrophages by FACS. The labeling index in the controls was <1% whereas around 10% of F4/80^+^ Kupffer cells were dual positive for the proliferative marker Ki67 and for BrdU labeling in the CSF1-Fc treated mice (**Figure 7c**). CSF1-Fc also produced a significant increase in splenic PCNA^+^ cells, probably reflecting the ability to drive extramedullary hemopoiesis^2^ (**Figure 7a**,**b**).

### Microarray data of liver gene expression

**Figure 8:**
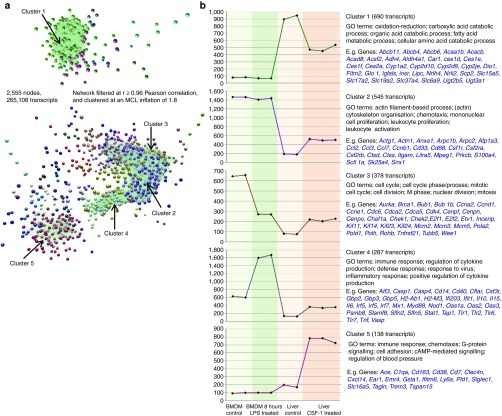
**Network analysis of liver transcripts post pig CSF1-Fc treatment.** Expression data for mouse livers +/- CSF1-Fc (*n* = 3) was analyzed alongside that of BMDM expression data (+/- LPS). (**a**) A network graph of transcript-to-transcript Pearson correlation relationships was filtered to show relationships of *r* ≥ 0.96, resulting in a graph of 2,555 nodes (transcripts) connected by 265,108 edges (Pearson correlation relationships). The graph was then clustered using the MCL clustering algorithm into groups of co-expressed genes. Nodes with the same color belong to the same cluster of co-expressed genes and tend to be highly connected within the network. (**b**) Expression data for five clusters with each point on the graph representing an individual mouse.

Gene expression arrays were used to determine whether there were any changes in liver function and whether there was expression of known hepatocyte growth factors. Of 2969 transcripts that were differentially expressed in the CSF1-Fc treated livers, 1020 genes were repressed by CSF1-Fc treatment and 1948 genes induced. To segregate these data into co-expressed gene sets that were likely to be expressed by liver cells versus macrophages, and genes potentially induced in the macrophages by exposure to LPS in the portal venous blood, the liver datasets were analyzed alongside data from BMDM cultured in CSF1, with or without stimulation with LPS.^25^ Network analysis of the normalized expression data was performed using BioLayout *Express*^3D^ as described previously.^25^ This generated a graph of 2,555 nodes representing distinct transcripts (**Figure 8a**). To identify groups of tightly co-expressed genes, the graph was clustered using the graph-based Markov clustering algorithm set at an inflation value (which determines the granularity of the clusters) of 1.8. This generated 45 clusters of co-expressed transcripts with membership sizes ranging from 690 to 4 nodes. **Figure 8b** shows the average expression profiles of the five largest clusters. Cluster 1 comprised of 690 transcripts collectively repressed in the livers of treated mice and absent or low-expressed in the BMDM samples. The gene ontology (GO) annotation indicated enrichment for intermediary metabolism, and the list includes numerous enzymes of glucose, amino acid, and lipid metabolism and cytochrome P450 members. The most interesting members of this cluster were the growth hormone receptor (*Ghr*), glucagon receptor (*Gcgr*), and insulin receptor (*Insr*), which could be linked to the changes in metabolism. Note that the repressed cluster did not contain liver-specific genes such as albumin (*Alb*), which indicates *inter alia* that the influx of macrophages is not sufficient to dilute the hepatocyte contribution to the total mRNA pool.

Cluster 2 (545 transcripts) contained genes expressed in BMDMs (regardless of LPS treatment) that were also inducible in CSF1-Fc treated livers. As anticipated, this cluster is clearly enriched in known macrophage-specific genes, including *Emr1* (F4/80), *Csf1r*, *Itgam* (CD11b), *CD68, lyz1* (lysozyme), and *Mpeg1* consistent with an increase in macrophage representation within the total mRNA pool of treated livers. The cluster also contains the chemokines *Ccl2, Ccl3*, and *Ccl7.* Clusters 3 and 6 also contained genes inducible in CSF1-Fc treated livers, but in contrast to cluster 2, these 378 transcripts were down-regulated by LPS in BMDMs. Both these clusters contain numerous cell-cycle associated genes, including *Pcna,* identified previously in cluster of a mouse gene expression atlas^26^; they may differ in the stages of the cycle they represent. Cluster 3 also contains the well-characterized CSF1 target gene, *Plau* (urokinase plasminogen activator).^27^ Their repression in the BMDM is consistent with the known ability of LPS to block CSF1 action and cause growth arrest in these cells.^28^ Finally, and importantly, there is a cluster of genes (Cluster 4) that is induced by LPS in the BMDM and also induced, to a lesser extent, in the CSF1-Fc treated livers. These include both pro-inflammatory (*Il1*, *Il6,* and *Tnf*), and anti-inflammatory (*Il10*) cytokines, several chemokines (*Cxcl10*, *Cxcl16*) and numerous known targets of interferon signaling (e.g. *Oas1, Gbp3, Ifit1, Irf7, etc.*) identified previously.^25,26^ Hence, the infiltration of the liver by macrophages in response to CSF1-Fc is accompanied by induction of classical proinflammatory cytokines. Interestingly, despite the expression of these genes, the inducible gene clusters did not include any known hepatocyte acute-phase gene products.

## Discussion

This study describes the biological efficacy of an Fc conjugate of CSF1. As shown in **Figure 1c**, the Fc addition to pig CSF1 increased the circulating half-life substantially without reducing biological efficacy measured on factor-dependent cells. There was formal possibility that the CSF1-Fc would trigger proinflammatory cytokine production by signaling through the Fc receptor, CD64^29^. However, the conjugate did not induce pro-inflammatory cytokine release *in vitro*. More importantly, *in vivo*, there was no evidence of pyrexia or weight loss in treated mice or pigs, nor any infiltration of the tissues by granulocytes indicative of acute tissue inflammation.

With the CSF1-Fc, daily injections can maintain a maximally active concentration, and even dosing every second day produced elevated monocyte numbers in the pig (**Supplementary Figure S1**). CSF1-Fc thus provides a novel reagent to study the role of macrophages in immunity, repair, and homeostasis.^2^ Using this novel new agent, we sought evidence for an effect of excess CSF1 on CSF1-dependent pathways identified from studies of the *op/op* mice. There is no evidence for the expression of the *Csf1r*–EGFP transgene within epithelia in any region of the gut.^5^ The intimate association of *Csf1r*-EGFP positive macrophages with the crypt epithelium (**Supplementary Figure S2a**) could produce indirect effects, but CSF1-Fc had no effect on epithelial architecture or Paneth cell numbers (**Supplementary Figure S2b**). By contrast, and in keeping with known dependence of osteoclasts upon CSF1, the administration of CSF1-Fc greatly increased their numbers within the epiphyseal plate (**Figure 6a**,**b**). This finding indicates that osteoclast numbers are sensitive to the availability of CSF1 in the steady state. There was no change in bone density or trabecular architecture in the short time frame examined. The treatment will not necessarily reduce bone density, because of the tight coupling of calcium homeostasis. In fact, Lloyd et al.^30^ found that bone density was increased in mice administered high-dose rh-CSF1 for 21 days and CSF1 can promote intramembranous ossification in a fracture model.^31^ These findings may reflect a separate role for the abundant population of CSF1-dependent macrophages that line bone surfaces.^32^ CSF1-Fc could therefore find applications in treatment of bone injury and osteoporosis.

The *op/op* mouse has deficiencies in both male and female fertility.^3^ Previous studies have described the close physical and functional relationship between TIMs and Leydig cells and the role of CSF1 in control of male fertility. CSF1 may also control testosterone production at the level of the hypothalamus and pituitary, by influencing the negative-feedback control of LH secretion.^17^ We demonstrated that CSF1-Fc increased TIM numbers and supported increased testosterone production, over and above the normal stimulation provided by pituitary LH (**Figure 4**). Furthermore, the observed increase in serum testosterone occurred in the absence of a change in circulating LH concentration. These data suggest that CSF1 acts both to support testosterone production at the level of the testis, and also to disrupt the negative-feedback mechanism at the level of the hypothalamus and pituitary. Our data indicate for the first time that the availability of CSF1 can control testosterone production in an adult animal. As such CSF1-Fc has a potential role as a gonadotropin-independent promoter of testicular testosterone production, with potential applications in medical and agricultural areas.

The most striking and unexpected effect of CSF1-Fc was the 50% increase in the size of the liver (**Figure 2c**). By comparison, there was a 15–37% increase in liver weight of mice infused with hepatocyte growth factor.^33^ Neither the *Csf1r*-EGFP transgene,^5^ nor *Csf1r* mRNA^34^ is expressed by hepatocytes. So, as in the context of renal regeneration and lung development,^35^ we favor an indirect effect via macrophage-expressed gene products. Broadly speaking, macrophages elicited by CSF1 treatment fall within the broad M2 or alternative classification of activation states,^35^ but the actions of CSF1 are quite distinct from those of the alternative activator, IL4^24^. Following removal of two-thirds of the liver, the remaining liver becomes hyperplastic and mature hepatocytes replicate to restore the original liver mass.^36^ As many as 95% of normally quiescent hepatocytes enter into the cell cycle in response to the priming cytokines (TNF*α* and IL-6) followed by progression through the cell cycle in response to growth factors such as hepatocyte growth factor, epidermal growth factor, and transforming growth factors.^36,37^ The treatment with CSF1-Fc largely mimics the effect of partial hepatectomy. The CSF1-dependent expression of urokinase plasminogen activator (*Plau*) may be a key mediator in the response to CSF1-Fc, since this protease is required for the release and activation of hepatocyte growth factor.^36^ Insulin, glucagon,^37^ and growth hormone^38^ have also been implicated in liver regeneration, and all three receptors were down-regulated in the livers of CSF1-Fc treated mice. Both TNF*α* and IL-6, which were induced in the liver of treated mice, have been identified as initiators of liver regeneration after partial hepatectomy based upon inhibitory effects of anti-TNF*α* antibodies^39^ and defective regeneration in mice lacking IL-6^40^. These cytokines are presumably induced through encounter with microbial products arriving in the portal circulation, but the levels are not sufficient to produce systemic effects (**Figure 1**), nor local infiltration of granulocytes, nor induction of classical acute-phase genes.

CSF1 is itself cleared from the circulation by the liver.^8^ Liver regeneration does not occur in the CSF1 deficient *op/op* mouse^41^ and is also prevented by macrophage depletion using toxic liposomes.^42,43^ Together, these observations indicate that CSF1-dependent macrophages are necessary for liver regeneration. We reported elsewhere that prolonged anti-CSF1R antibody treatment can reduce the size of the liver.^4^ The surprising result herein is that CSF1-Fc treatment alone is sufficient to promote proliferation in an undamaged liver. The impact of CSF1-Fc treatment indicates that there is a simple feedback loop between circulating CSF1 availability, monocyte production and release by the BM, monocyte extravasation, and the size of the liver. The remarkable effect of CSF1-Fc on the liver (**Figure 7**) has clear implications for regenerative therapy. We reported previously that administration of CSF1-stimulated macrophages via the portal vein can promote repair in chronic liver injury.^44^ Our current data suggest that the same outcome may be achieved by the administration of the agonist alone without adverse effects.

In other regenerative medicine applications, increasing the size of the liver may, or may not, be desirable. It could improve the clearance of agents that mediate the pathology. The Fc conjugate will certainly improve the efficacy and viability of CSF1 as a treatment in the many other organ systems in which it has already been shown to have a beneficial effect^2^ and others such as heart^45^ and spinal cord,^46^ where macrophages are crucial to repair. Systemic CSF1 administration was recently found to be effective in protection against acute brain injury^47^ and promoted the clearance of beta-amyloid deposits in a mouse model of Alzheimer's disease.^48^ The CSF1-Fc provides a new and significantly more cost-effective reagent to extend these preclinical studies to large animals. We have established transgenic chicken lines that produce the protein in eggs to produce the large amounts of protein required for these studies (unpublished). An additional advantage of the Fc tag is that the protein can also be made using standard commercial protein expression, and readily purified by protein A affinity chromatography. Clearly applications in humans and companion animals will require the production of the appropriate species-specific reagents, but the pig offers many opportunities for preclinical evaluation.

Nevertheless, our data also demonstrate that treatment also has pleiotropic effects, most of which would be predicted from the complex phenotype of the *op/op* mouse. The array data indicate a global effect on hepatocyte lipid and amino acid metabolism and on responsiveness to insulin and glucagon. Taking all of these findings together, CSF1 emerges as a central homeostatic regulator. Accordingly, therapeutic applications will need to proceed with caution.

## Materials and Methods

***Cloning and expression of pig CSF1-Fc.*** The sequence corresponding to the active fragment of pig CSF1 (SENCSHMIGDGHLKVLQQLIDSQMETSCQIAFEFVDQEQLTDPVCYLKKAFLQVQDILDETMRFRDNTPNANVIVQLQELSLRLNSCF TKDYEEQDKACVRTFYETPLQLLEKIKNVFNETKNLLKKDWNIFSKNCNNSFAKCSSQHERQPEGR) was linked to the hinge-CH3 region of the pig IgG1a sequence (GTKTKPPCPICPGCEVAGPSVFIFPPKPKDTLMISQTPEVTCVVVDVSKEHAEVQFSWYVDGVEVHTAETRPKEEQFNSTYRVV SVLPIQHQDWLKGKEFKCKVNNVDLPAPITRTISKAIGQSREPQVYTLPPPAEELSRSKVTVTCLVIGFYPPDIHVEWKSNGQPEPE GNYRTTPPQQDVDGTFFLYSKLAVDKARWDHGETFECAVMHEALHNHYTQKSISKTQGK). This entire region was codon optimized for mammalian expression by GeneArt (Invitrogen, Paisley, UK) and cloned into the expression plasmid pS00524 using HindIII and NotI restriction sites engineered into the 5′ and 3′ ends respectively. The resulting plasmid was sequenced to ensure ORF integrity and protein was expressed from transfected HEK293F or CHO cells.

***Isolation of pig CSF1-Fc fusion protein.*** Pig CSF1-Fc fusion protein produced under contract by Genscript Inc. Briefly, it was isolated using Protein A affinity chromatography. Briefly, conditioned medium from cell culture was centrifuged and loaded onto Protein A Sepharose that was equilibrated with PBS. Following loading, the column was washed with PBS and 35 mmol/l Na Acetate pH 5.5. Protein was eluted using a step gradient of 80% B Buffer (35 mmol/l Acetic acid, no pH adjustment), 85% B buffer, and 100% B buffer. The 80 and 85% B fractions were pooled based on lack of aggregated protein (analytical SEC) and the 100% B fraction was not included. Pooled protein was pH adjusted to 7.2 and dialyzed against PBS. Purity was assessed by mass spectrometry, and endotoxin was certified less than 10 EU/mg. The protein migrated as a single band of the expected Mr (85-90kD) on SDS-PAGE, and of 45–50kD under reducing conditions.

***Cell viability assay.*** Stable Ba/F3 cells expressing pig CSF1R^14^ were maintained in culture with complete RPMI supplemented with 10^4^ Units/ml rh-CSF1 prior to MTT assay. 2 × 10^4^ cells/well (Ba/F3 cells, or 5 × 10^4^ cells/well (pig BMM) of a 96 well plate were plated in triplicate or quadruplicate and treated (serial dilutions of pig CSF1 or CSF1-Fc were added to make a total volume of 100 μl per well. Cells were incubated for 48 hours at 37 °C, 5% CO_2_. For Ba/F3pCSF1R cells, 10 μl of MTT (Sigma Aldrich, St Louis, MO M5655) stock solution (5 mg/ml) was added directly to each well to achieve a final concentration of 0.5 mg/ml and incubated at 37 °C for 3 hours prior to solubilization overnight. For adherent pig BMM cells, the culture medium was replaced with 50 µl of 1 mg/ml MTT solution and incubated for 1 hour at 37 °C. The MTT solution was removed and tetrazolium salt solubilized with 100μl of solubilization agent (0.1 M HCL, 10% Triton X-100 and isopropanol) followed by incubation at 37 °C with 5% CO_2_ for 10 minutes. Plates were read at 570 nm with reference wavelength of 405 nm.

***Pig CSF1 and CSF1-Fc ELISA.*** Pig CSF1-Fc-fusion plasma levels were detected using an ELISA, developed in-house using human anti-CSF1 antibody (Abcam, ab9693) at 0.3 µg/mL and rabbit anti-pig IgG (Fc) biotinylated antibody (Alpha Diagnostic, 90440) at 1:5000 dilution. Standard protein was generated and purified in-house by Zoetis. Standards were added to each plate along with the samples resulting in an 11-point standard range of 2700 ng/mL–0.046 pg/mL. This allowed for quantitation of each sample to a standard curve on every assay plate. Assay detection was done using Pierce High Sensitivity Streptavidin-HRP (1:10,000 dilution) and TMB Microwell Peroxidase Substrate System solution (KPL).

***Pig CSF1-Fc pharmacokinetic study.*** Weaner age barrows (castrated male) (<14 kg) were assigned to three treatment groups receiving a single intravenous (i.v.) or subcutaneous (s.c.) dose as follows: 0.5 mg/kg CSF1 s.c. (*n* = 3), 1.2 mg/kg CSF1-Fc i.v. (*n* = 2) or 1.2 mg/kg CSF1-Fc s.c. (*n* = 2). Blood was collected from the jugular into EDTA tubes followed by separation of plasma. Serial plasma samples were obtained from each animal at predose and 5 minutes, 30 minutes and 1, 2, 4, 6, 8, 24, 48, and 72 hours postdose. CSF1 and CSF1-Fc plasma protein concentrations were quantitated using ELISA assays.

**In vivo *mouse study.*** *Csf1r*-EGFP^+^ (MacGreen), *Csf1r*-EGFP^-^ wild-type mice were bred, genotyped, and obtained from The Roslin Institute Biological Research Facility. C57BL/6 mice were purchased from Charles River Laboratories (UK). Approval was obtained from The Roslin Institute's and The University of Edinburgh's Protocols and Ethics Committees. The experiments were carried out under the authority of a UK Home Office Project Licence under the regulations of the Animals (scientific procedures) Act 1986. Male and female 6–10 week MacGreen (*n* = 8 per sex), and C57BL/6 mice (*n* = 12 per sex), were injected subcutaneously with either PBS (*n* = 24) or 1 mg/Kg pig CSF1-Fc (*n* = 24) for 4 days prior to sacrifice on day 5. Mice were weighed every day prior to injection and before cull. One mouse from each treatment group was tissue perfused with 4% PFA. Blood was collected by cardiac puncture.

***Bone marrow cells.*** Bones were stripped carefully of any tissue/muscle placed in 70% ethanol for 1 minute prior to PBS for 1 minute. The proximal and distal ends of the femur were cut and cells flushed with RPMI. Cells were re-suspended prior to centrifugation at 400*g* for 5 minutes and counted. Cells for FACS analysis were harvested as above and prepared at a concentration of 5 × 10^6^ cell/sample.

***Fluorescence-activated cell sorting.*** BM cells were washed, pelleted, and re-suspended in PBS containing 0.1% NaN_3_, 2% heat inactivated FCS, 0.1% BSA) with 2% heat inactivated normal mouse serum (200 µl/sample), and incubated on ice for 20 minutes. Following washes (PBS containing 0.1% NaN_3_, 0.2% heat inactivated FCS, 0.1% BSA: Lo-block), cells were re-suspended in the appropriate antibody or isotype control (1:200 of PE anti-mouse F4/80 Biolegend 122616, 1:200 PE anti-mouse Ly-6G/Ly-6C (Gr-1) Biolegend 108408 or 1:200 PE Rat IgG2b, k isotype control Biolegend 400608) and incubated for 30 minutes. Cells were re-suspended in 400 µl of Lo-block with 0.1% Sytox Blue (Invitrogen). Samples were analyzed on CyAn (Dako, Ely, UK) and analyzed using Summit 4.1 software (Dako).

The liver was perfused via the inferior vena cava and portal vein with PBS and then digested in 2 mg/ml collagenase D (Sigma Aldrich) at 37 °C for 30 minutes and passed through a 100 μm filter. A 7 minutes, 50G spin was performed to remove hepatocytes. Nonparenchymal cells were further purified using a 30% percoll (Sigma) gradient. Cells were stained with fixable viability die eFluor 780 then incubated with Fc block (TrustainfcX, Biolegend) prior to staining with CD45 (AF700, Biolegend) and F480 (PECy7, Biolegend). Cells were fixed and permeablized using BD Pharminogen BRDU flow kit then stained with antiBRDU (FITC, BD Pharminogen) and Ki67 (eF660, eBioscience). Flow cytometry was performed using the LSR Fortessa.

***Tissue processing.*** Tissues from perfusion-fixed *Csf1r*-EGFP^+^ mice were placed into 4% PFA for 2 hours followed by overnight incubation in 18% sucrose at 4 °C. The following day, tissues were embedded in Tissue-Tek OCT compound and frozen in isopentane. Frozen sections were cut (8–12 um thick) at −16 °C using LEICA cryostat and mounted with Dako fluorescent mounting medium (Dako). The fluorescence of the EGFP was visualized using Zeiss confocal and pictures taken under oil at 400x magnification. Additional tiled images composed of nine images as above were also generated. The total fluorescence was analyzed using Image J. Tissues from non-perfused mice were weighed and placed in 10% formal saline and RNALater. The left femur after thorough muscle and tissue stripping was placed in formalin at room temperature overnight followed by transfer into 70% ethanol the following day.

***Immunohistochemistry.*** For tissue histology, tissues were processed using Excelsior tissue processor (Thermo Fisher Scientific). Sections were then placed in moulds, embedded in paraffin wax 4 µm sections cut, and mounted onto slides (Superfrost Plus, Thermo Fisher Scientific). Antigen retrieval was performed in Proteinase K (Dako S302030) for 10 minutes at 25 °C. Non-specific protein binding was blocked using 2.5% goat serum for 20 minutes at room temperature (Vector Laboratories). Endogenous peroxidase activity was blocked using Dako REAL peroxidase blocker (Dako REAL blocking agent S202386) for 10 minutes following antibody incubations. Sections were incubated at room temperature for 30 minutes using rat anti-F4/80 (Serotec MCA497G) diluted 1/400 in TBST. Negative controls were carried out using rat IgG at the same concentration as the primary antibody. Visualisation was carried out using the secondary reagent Immpress anti rat HRP (Vector Labs MP-7444-15) for 15 minutes at room temperature followed by DAB (Newmarket Scientific Monosan Dab substrate kit Cat No. MON-APP177) for 10 minutes and DAB enhancer for 3 minutes (Newmarket Scientific DAB concentrate Cat No.CO7-25). For Proliferating Cell Nuclear Antigen sections were prepared as above on poly-L-lysine coated slides with antigen retrieved by boiling 10 mmol/L sodium citrate buffer followed by blocking for endogenous peroxidase activity with 3% H_2_O_2_ in methanol for 10 minutes. Immunohistochemistry was performed using PCNA staining kit (Invitrogen, 93–1143) as per manufacturer's instructions.

Mouse femurs were decalcified using 14% EDTA (pH7) for 3 days at room temperature, dehydrated, and embedded in wax for cutting of sections. TRAP^+^ cells were identified using an Acid Phosphatase kit (Sigma-Aldrich 387) according to manufacturer's instructions.

For detection of lysozyme in intestinal crypts, 4% paraformaldehyde-fixed sections were permeabilized with 50% methanol for 20 minutes before immunostaining with anti-lysozyme monoclonal antibody. Appropriate species and immunoglobulin isotype control antibody were used as controls. Species-specific Alexa-Fluor 594 (red; Invitrogen) secondary antibody dye was used and sections were counterstained with Alexa-647 Phalloidin (blue; Dako). Sections were mounted in fluorescent mounting medium (Dako) and examined using a Zeiss LSM5 confocal microscope (Zeiss, Welwyn Garden City).

For detection of macrophages in the testis, samples were fixed in 4% paraformaldehyde at 4 °C for 24 hours, and then moved to PBS, prior to analysis. Sections were deparaffinized, rehydrated, and antigen retrieved using a citrate buffer epitope retrieval method (10 Psi (0.68 atm), 125 °C, 30 minutes, in citrate buffer pH 6.0) before blocking of endogenous peroxidase and non-specific binding sites. Anti-Mac 2 (Cedarlane labs, Cat. No. CL8942) was applied at 1:500 in normal horse serum, and incubated at 4 °C for 24 hours, followed by incubation for secondary detection (Vector, immpressTM, Reagent Kit, Cat. No. MP-7401), for 1 hour. Samples were washed in TBS and DAB detection (Vector, ImmPACT Peroxidase Substrates, Cat. No. SK-4105) was used to resolve sites of immunolocalization, while hematoxylin was used as counterstain. Sections were then mounted for downstream analysis and visualized using an Olympus Research Microscope (AX70 Provis, Scotia, NY, USA, software: AxioVision Rel.4.8).

***Hormone assays.*** Quantification of circulating hormones was carried out as previously described.^49,50^

***Statistical analysis.*** Data on body weight changes, complete blood counts, organ weights, CSF1, CSF1-Fc, and IGF-1 ELISA were analyzed using a Mann–Whitney test or Kruskal–Wallis test with Dunn's multiple comparison test. Results are presented as either individual value with the mean ± standard error of the mean (SEM) or group mean ± SEM. All analyzes were performed using GraphPad Prism 5.0 (GraphPad Software Inc, San Diego, CA). A *P* value < 0.05 was considered statistically significant.

***Microarray.*** RNA was extracted from mouse liver using RNA Bee as per manufacturer's instructions. RNA integrity and quality was assessed using Following the RNA 6000 LabChip Kit (Agilent). Samples with a RNA integrity number greater than seven was used for microarray. Microarray was performed by ARK Genomics, The Roslin Institute. Total RNA of 50 ng was amplified using the Nugen Pico SL kit, 2.5 ug of the resulting cDNA was biotin labeled using the Nugen Encore labeling kit using the half volume protocol. The biotin labeled product was prepared for hybridisation according to the Nugen protocol for Affymetrix Gene Titan hybridisation, using the Affymetrix Gene Titan Hybridization Wash & Stain Kit for WT Array Plates (PN 901622). The samples were hybridized to Affymetrix Mouse Gene 1.1 ST Array plates using the appropriate Hyb-Wash-Scan protocol for this plate and the Gene Titan Hyb Wash Stain kit for the reagents (Affymetrix). Image generation and the resulting CEL files for analysis were produced in Affymetrix^®^ GeneChip^®^ Command Console^®^ Software (AGCC) version 3.0.1. Initial QCs were performed in Expression Console. The obtained AffymetrixCEL files were imported into the Genomics Suite software package version 6.13.0213 (Partek software. Copyright, ©2012. Partek Inc.)

Expression data from the liver +/− CSF1-Fc study was analyzed alongside that of BMDM data generated from the same strain of mice (C57BL/6) (see BMDM method below). A network analysis of the normalized expression data was performed using BioLayout *Express*^3D^. (www.biolayout.org) Using this tool, a Pearson correlation matrix of a transcript-to-transcript profile comparison was used to filter for expression correlation relationships of *r* ≥ 0.96 across the microarray samples. Nodes within the network graph represent transcripts and the edges between them represent expression correlations above the set threshold (*r* ≥ 0.96 in this case). To identify groups of tightly co-expressed genes, the graph was clustered using the graph-based clustering algorithm MCL set at an inflation value (which determines the granularity of the clusters) of 1.8. Gene lists associated with the clusters were exported for GO annotation analysis (Biological and Metabolic Processes Level-FAT) using the DAVID (Database for Annotation, Visualization and Integrated Discovery) tool. GEO accession No. GSE52636.

***BMDM production for microarray.*** BMDM were prepared as above from C57BL/6J mice and cultured for 6 days in RPMI 1640 (Sigma-Aldrich) supplemented with 10% heat inactivated fetal bovine serum (Sigma-Aldrich), 25 U/ml penicillin (Invitrogen), 25 μg/ml streptomycin (Invitrogen), and 2 mmol/l l-glutamine (Invitrogen) and 10,000 U/ml CSF1 on 10 cm^2^ bacteriological plastic plates. On day 6, cells were harvested, counted, re-suspended in complete medium with 10,000 U/ml CSF1, and seeded into 24-well tissue culture plates at a density of 200,000 cells/well. After 24 hours, (day 7) C57BL/6 derived macrophages were treated with 50 ng/ml LPS at harvested 8h post-treatment, or harvested as untreated (control) macrophages. GEO accession No. GSE44292

**SUPPLEMENTARY MATERIAL** **Figure S1.** Effect of pig CSF1-Fc on piglet growth and WBC counts. **Figure S2.** Effect of pig CSF1-Fc on intestine.

## Supplementary Material

Supplementary Figure S1 Effect of pig CSF1-Fc on piglet growth and WBC counts.

Supplementary Figure S2 Effect of pig CSF1-Fc on intestine.
