# Supplementary figures and images for: Characterisation of a Novel Fc Conjugate of Macrophage Colony-stimulating Factor
Source: Mol Ther. 2014 Jul 29;22(9):1580–92. doi: 10.1038/mt.2014.112 (PMC4435485; doi:10.1038/mt.2014.112)

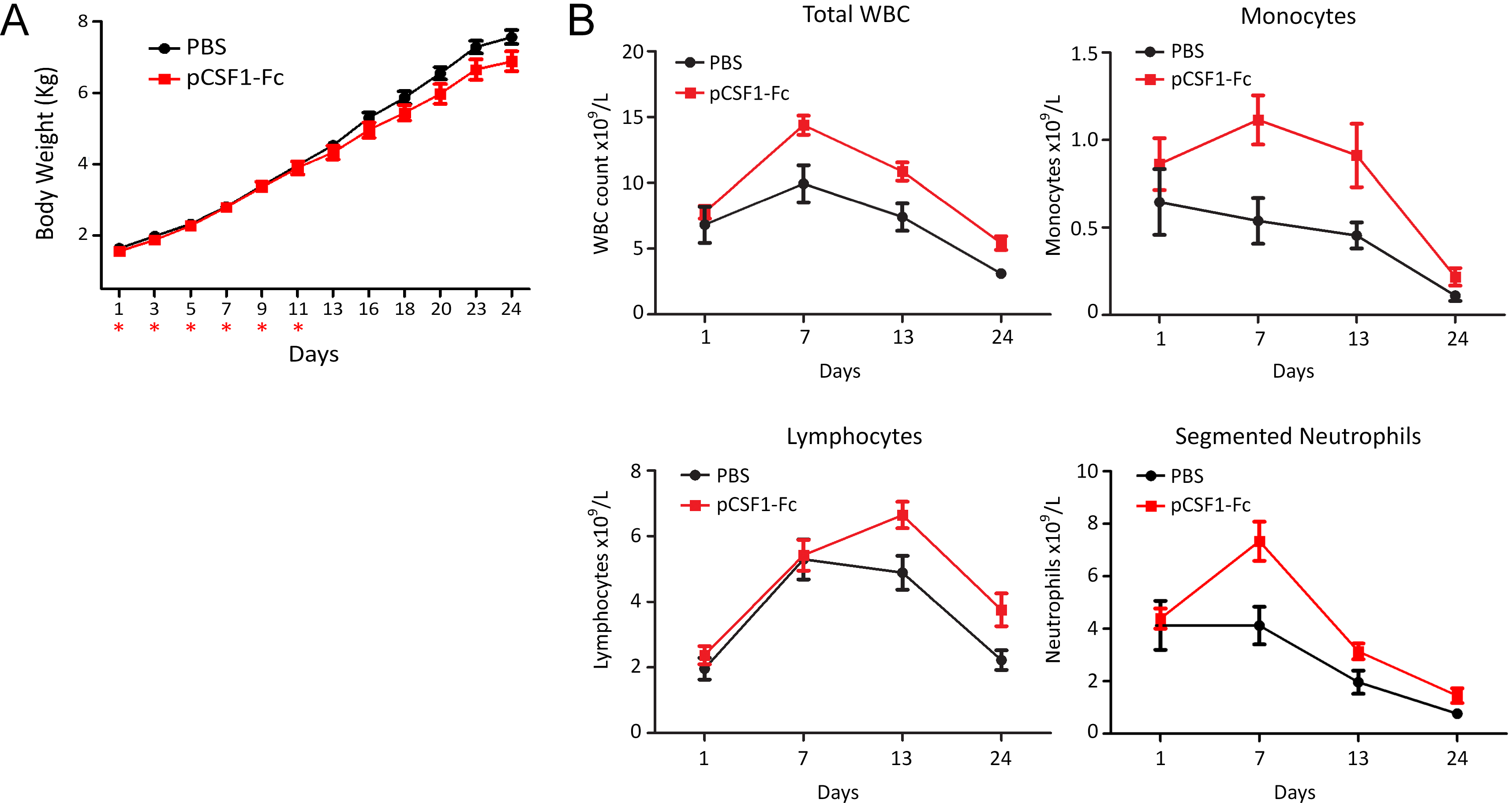

Supplement: Supplementary Figure S1 — Effect of pig CSF1-Fc on piglet growth and WBC counts. [file mt2014112x1.tiff]

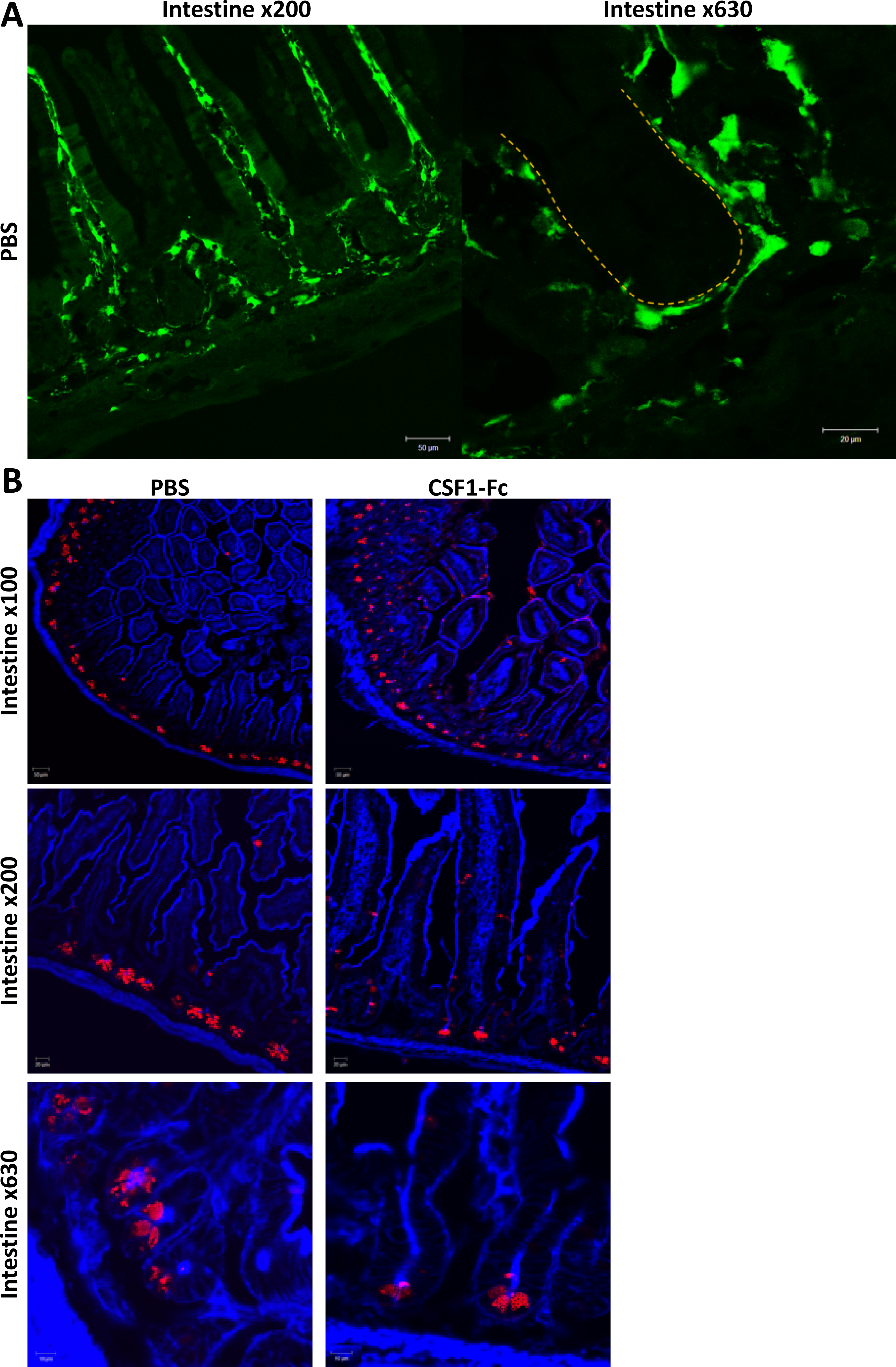

Supplement: Supplementary Figure S2 — Effect of pig CSF1-Fc on intestine. [file mt2014112x2.tiff]
